# Generalized Orthogonal de Bruijn and Kautz Sequences †

**DOI:** 10.3390/e27040366

**Published:** 2025-03-30

**Authors:** Yuan-Pon Chen, Jin Sima, Olgica Milenkovic

**Affiliations:** Department of Electrical and Computer Engineering, University of Illinois Urbana-Champaign, Urbana, IL 61801, USA; jsima@illinois.edu (J.S.); milenkov@illinois.edu (O.M.)

**Keywords:** De Bruijn sequences, orthogonal de Bruijn sequences, balanced de Bruijn sequences, fixed-weight de Bruijn sequences, Kautz sequences, orthogonal Kautz sequences, synthetic biology

## Abstract

A de Bruijn sequence of order *k* over a finite alphabet is a cyclic sequence with the property that it contains every possible *k*-sequence as a substring exactly once. Orthogonal de Bruijn sequences are the collections of de Bruijn sequences of the same order, *k*, that satisfy the joint constraint that every (k+1)-sequence appears as a substring in, at most, one of the sequences in the collection. Both de Bruijn and orthogonal de Bruijn sequences have found numerous applications in synthetic biology, although the latter remain largely unexplored in the coding theory literature. Here, we study three relevant practical generalizations of orthogonal de Bruijn sequences, where we relax either the constraint that every (k+1)-sequence appears exactly once or the sequences themselves are de Bruijn rather than balanced de Bruijn sequences. We also provide lower and upper bounds on the number of fixed-weight orthogonal de Bruijn sequences. The paper concludes with parallel results for orthogonal nonbinary Kautz sequences, which satisfy similar constraints as de Bruijn sequences, except for being only required to cover all subsequences of length *k* whose maximum run length equals one.

## 1. Introduction

Parts of the work have been submitted to the IEEE Symposium on Information Theory (ISIT), Ann Arbor, MI, USA, 2025 [1]. This extension contains added proofs for results on orthogonal de Bruijn sequences and a completely new section on orthogonal Kautz sequences.

De Bruijn sequences [2,3] are combinatorial objects that have found many practical applications, which range from pesudorandomness generation, hashing, and lookup table design to DNA assembly and molecular data storage [4]. The utility of the de Bruijn sequences of order *k* stems from the fact that they have the property of covering all *k*-sequences over a finite alphabet as substrings exactly once. De Bruijn sequences have been further generalized to include *balancing constraints* [5]—in which case, every *k*-sequence is allowed to appear *ℓ* ≥1 or at most *ℓ* times—or general substring constraints, as described in [6]. In addition, they have been modified to accommodate other practical constraints, such as run length bounds, in which case, the sequences are known as *Kautz sequences* [7].

Another interesting extension of the concept of de Bruijn sequences is *orthogonal de Bruijn sequences*, introduced in [8] for the purpose of optimizing experimental designs in synthetic biology (they were also independently studied in the mathematics literature [9,10] under the name of arc-disjoint de Bruijn cycles). In a nutshell, orthogonal de Bruijn sequences are the de Bruijn sequences of order *k* that satisfy the joint (cross) property, where every (k+1)-sequence appears in at most one of the sequences in the collection. The de Bruijn property of the sequences is used to ensure both the *diversity of DNA sequence binding probes* of length *k* and the shortest sequence length property under the diversity constraint (since DNA strings have to be synthesized for testing and since the cost of synthesis prohibits the use of long strings). Interestingly, the orthogonality constraint aims to reduce the undesired cross-hybridization of longer probes designed to target only one of the sequences; although, in the definition, the constraint is imposed on length-(k+1) substrings, other constrained substring lengths (such as 2k) are equally relevant. From the perspective of DNA-based data storage, orthogonal de Bruijn sequences are relevant for multistage primer-based random access [11]. There, groups of strings sharing a common *k*-substring can be accessed together (for any possible choice of the substring), and then further partitioned into subgroups as needed, using more specialized primers that are not shared by the whole group (say, using primers of length k+s, s>0). A drawback of orthogonal de Bruijn sequences is that their number is strongly restricted by the alphabet size. We can increase an orthogonal collection by relaxing the notion of orthogonality, as described in Section 3 (following the preliminaries of Section 2). There, we study orthogonal de Bruijn sequences in which (k+1)-sequences are allowed to appear at most *ℓ* times, with *ℓ* ≥1. The main result is that the number of generalized orthogonal de Bruijn sequences scales with *ℓ*.

Another practical issue with orthogonal de Bruijn sequences is that each *k*-sequence has only one context in which it appears in each of the sequences. To increase the number of different contexts, we can examine *b*-balanced de Bruijn sequences in which each *k*-sequence is allowed to appear exactly *b* times [5]. In this case, we can investigate the (new) notion of orthogonality of balanced de Bruijn sequences, as outlined in Section 4. For ease of synthesis, it is desirable to maintain composition/weight constraints for the DNA sequences, resulting in counting and construction problems pertaining to fixed-weight (fixed composition) de Bruijn sequences, which are introduced and studied in Section 5.

We conclude our exposition with a review of Kautz and orthogonal Kautz sequences and the introduction of balanced and fixed-weight Kautz and orthogonal Kautz sequences, as described in Section 6. The relevance of the added run length constraint is that the sequences avoid what is known as *homopolymers* of DNA symbols, which are known to cause both DNA synthesis and sequencing errors. This is particularly the case for nanopore sequencers, as first described and experimentally evaluated in [12].

## 2. Preliminaries

We start by reviewing relevant concepts and definitions pertaining to (orthogonal) de Bruijn sequences and graphs.

**Definition 1.**  *Let k≥1, and let A be an alphabet of size σ≥2. A circular sequence $s=(s_{0},s_{1},\ldots ,s_{\sigma^{k}-1})$ is called a* (σ,k)-de Bruijn sequence *if each sequence in $A^{k}$ appears as a circular substring of s exactly once. More specifically, for each sequence $t=\left(t_{0},\ldots ,t_{k-1}\right)\in A^{k}$, there is a unique index $i\in [0,\sigma^{k}-1]$ such that $(s_{i},s_{i+1\;\mathrm{mod}\;\sigma^{k}},\ldots ,s_{i+k-1\;\mathrm{mod}\;\sigma^{k}})=t$. Here, we used [a,b] to denote the integer set {a,a+1,…,b} for two integers a and b that satisfy a≤b. We do not distinguish sequences that are circular shifts of each other.*

Fundamental for the study of de Bruijn sequences is the notion of a *de Bruijn graph* of order *k* and alphabet size σ, which is denoted by $G_{\sigma ,k}$. A de Bruijn graph is a directed graph (V,A) with vertex set $V=A^{k-1}$ and arc set

(1) $$\begin{array}{c} A=\{\left(\left(s_{0},s_{1},\ldots ,s_{k-2}\right),\left(s_{1},\ldots ,s_{k-2},s_{k-1}\right)\right)\;|\;s_{0},\ldots ,s_{k-1}\in A\}. \end{array}$$

In words, for $v_{1},v_{2}\in V$, there exists an arc from $v_{1}$ to $v_{2}$ if and only if the length-(k−2) suffix of $v_{1}$ is the same as the length-(k−2) prefix of $v_{2}$.

**Definition 2.**  *A collection of (σ,k)-de Bruijn sequences $C=\{s_{1},\ldots ,s_{M}\}$ is called* orthogonal *if each sequence in $A^{k+1}$ appears at most once in C.*

It is clear that there exists a one-to-one correspondence between (σ,k)-de Bruijn sequences and Eulerian circuits (i.e., closed walks that traverse each arc exactly once) in $G_{\sigma ,k}$. Furthermore, a length-(k+1) string $\left(s_{0},\ldots ,s_{k}\right)$ appears in a (σ,k)-de Bruijn sequence if and only if the corresponding Eulerian circuit traverses from $v_{1}$ to $v_{2}$ to $v_{3}$, where $v_{1}=\left(s_{0},\ldots ,s_{k-2}\right)$, $v_{2}=\left(s_{1},\ldots ,s_{k-1}\right)$, and $v_{3}=\left(s_{2},\ldots ,s_{k}\right)$.

Similarly, there exists a one-to-one correspondence between (σ,k)-de Bruijn sequences and Hamiltonian cycles (i.e., closed walks that traverse each vertex exactly once) in $G_{\sigma ,k+1}$. The length-(k+1) string $\left(s_{0},\ldots ,s_{k}\right)$ appears in a (σ,k)-de Bruijn sequence if and only if the corresponding Hamiltonian cycle traverses from $\left(s_{0},\ldots ,s_{k-1}\right)$ to $\left(s_{1},\ldots ,s_{k}\right)$.

The relevance of de Bruijn graphs $G_{\sigma ,k+1}$ for the analysis of orthogonal de Bruijn sequences comes from the fact that certain arc-constrained Hamiltonian cycles correspond to orthogonal sequences. In that context, it was shown in [8] that for σ≥3, the number of orthogonal (σ,k)-de Bruijn sequences is bounded between ⌊σ/2⌋ and σ−1.

**Figure 1:**
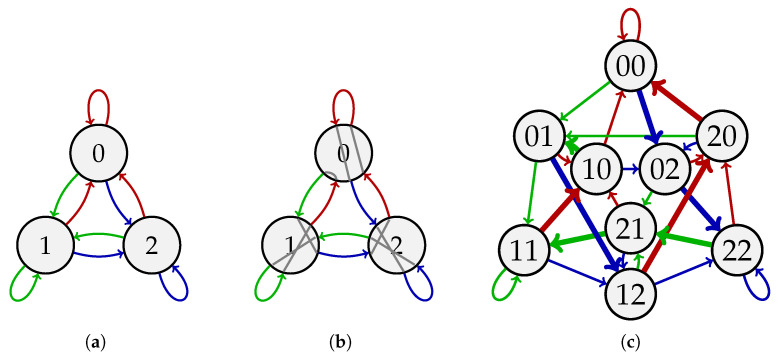
(**a**) The de Bruijn graph $G_{3,2}$. (**b**) An Eulerian circuit in $G_{3,2}$ that represents the (3,2)-de Bruijn sequence 012002211. (**c**) The de Bruijn graph $G_{3,3}$. The arcs in the Hamiltonian cycle of the sequence 012002211 are depicted with bold lines.

**Example 1.**  *Let k=2 and A={0,1,2}. The circular sequence s=012002211 is a (3,2)-de Bruijn sequence since its length-2 substrings are 01,12,20,00,02,22,21,11,10—all the length-2 sequences over A without repetition. Figure 1a shows the de Bruijn graph $G_{3,2}$ used to generate the sequence. Figure 1b depicts the Eulerian circuit on $G_{3,2}$ that s corresponds to, which is 0→1→2→0→0→2→2→1→1→0. Figure 1c illustrates the de Bruijn graph $G_{3,3}$, where s corresponds to the Hamiltonian cycle 01→12→20→00→02→22→21→11→10→01. The length*-3 *substring* 012 *in s corresponds to the two-step walk 0→1→2 in the Eulerian cycle on $G_{3,2}$ and the arc 01→12 in the Hamiltonian cycle on $G_{3,3}$.*

We also review the balanced de Bruijn sequence studied in [5].

**Definition 3**  ([5] Definition 4)**.** *Let k and A be as before, and let b≥1. A circular sequence $s=(s_{0},s_{1},\ldots ,s_{b\sigma^{k}-1})$ is called a b*-balanced (σ,k)-de Bruijn sequence *if each sequence in $A^{k}$ appears as a circular substring of s exactly b times.*

## 3. Generalized Orthogonal de Bruijn Sequences

Our first result pertains to a generalization of orthogonal de Bruijn sequences, defined below.

**Definition 4.**  *Let ℓ ≥1. A collection of (σ,k)-de Bruijn sequences $C=\{s_{1},\ldots ,s_{M}\}$ is called ℓ*-orthogonal *if each sequence in $A^{k+1}$ appears at most ℓ times in C.*

Let $\Omega_{\ell }\left(\sigma ,k\right)$ denote the maximum cardinality of a collection of *ℓ*-orthogonal (σ,k)-de Bruijn sequences. We have the following bound on $\Omega_{\ell }\left(\sigma ,k\right)$:

**Proposition 1.**  *We have $\Omega_{\ell }\left(\sigma ,k\right)\leq \ell \left(\sigma -1\right)$.*

**Proof.**  The proof follows a similar argument as in [8] Corollary 4. A collection of *ℓ*-orthogonal (σ,k)-de Bruijn sequences corresponds to a collection of Hamiltonian cycles in $G_{\sigma ,k+1}$ such that each arc is used at most *ℓ* times. Note that the all-zero vertex $0^{k}$ in $G_{\sigma ,k+1}$ (assuming A=[0,σ−1]) has σ−1 incoming arcs, σ−1 outgoing arcs, and a loop. Note that for a directed graph *G* and a vertex *v* of *G*, an incoming arc of *v* refers to an arc of the form (w,v), for some w≠v, while an outgoing arc of *v* refers to an arc of the form (v,x), for some x≠v; a loop on *v* is an arc (v,v). Also note that a Hamiltonian cycle cannot involve a loop. Therefore, by the pigeonhole principle, any collection of more than *ℓ* (σ−1) Hamiltonian cycles on $G_{\sigma ,k+1}$ uses at least one of the σ−1 incoming arcs of $0^{k}$ more than *ℓ* times. □

**Theorem 1.**  *If *ℓ* $\leq \sigma^{k-1}$ and σ≥3, then*

$$\Omega_{\ell }\left(\sigma ,k\right)\geq 2\ell ;\;\;\;\Omega_{\ell }\left(\sigma ,k\right)\geq \ell \left\lfloor \sigma /2\right\rfloor .$$

This result is intuitively expected, despite its proof being nontrivial. Before proceeding with the proof, we introduce the concept of “wiring” described in [8,9].

**Definition 5.**  *Let G be a directed graph with an Eulerian circuit C, and let v be a vertex in G. For a graph to be Eulerian, each vertex must have the same in-degree and out-degree, so that its incoming arcs and outgoing arcs are paired up in the circuit. A* wiring *of v [8], or a* transition system *at v [9], is a* 1*-regular bipartite undirected graph (i.e., a matching) between two vertex sets representing the incoming arcs and outgoing arcs of v. More precisely, the wiring of v induced by C, denoted by W(v,C), is a wiring such that an incoming arc $a_{i}$ connects to an outgoing arc $a_{o}$ if and only if the Eulerian circuit C uses $a_{i}$ and $a_{o}$ in consecutive order. In the case that v has a self-loop, we treat that loop as both an incoming arc and an outgoing arc.*

We say two Eulerian circuits are *compatible* [9] if the induced wirings are edge-disjoint at each vertex, i.e., two Eulerian circuits are compatible if they do not use the same in/out arc pair at any vertex. This leads to the following characterization of orthogonal de Bruijn sequences: A collection of (σ,k)-de Bruijn sequences is orthogonal if and only if their corresponding Eulerian circuits in $G_{\sigma ,k}$ are pairwise compatible.

In our proof of Theorem 1, we will make use of the following lemmas regarding wirings.

**Lemma 1**  ([8] Theorem 7)**.** *Let G=(V,A) be a directed graph with an Eulerian circuit C and let v∈V. Let deg(v) denote the in/out-degree of v (i.e., the in-degree of v, which is equal to the out-degree of v). If deg(v)≥3, there exists an Eulerian circuit $C^{\prime }$, denoted by $C^{\prime }\left(v,C\right),$ such that*

- *$W(v,C^{\prime })$ and W(v,C) are edge-disjoint;*
- *$W\left(v^{\prime },C^{\prime }\right)=W\left(v^{\prime },C\right)$ for all $v^{\prime }\in V\setminus \left\{v\right\}$.*

In words, we can rewire *v* with respect to *C* to obtain $C^{\prime }$ so that $C^{\prime }$ does not use the same in/out arc pair of *v* as *C*; the wirings at other vertices in $C^{\prime }$ remain the same as in *C*.

**Lemma 2**  ([8] Theorem 8; [9] Theorem 1)**.** *Let G=(V,A) be an directed graph with an Eulerian circuit C, and let $C_{1},C_{2},\ldots ,C_{t}$ be compatible Eulerian circuits of G. Furthermore, let v∈V. If t≤⌊deg(v)/2⌋−1, then there exists an Eulerian circuit $C^{\prime }$, denoted by $C^{\prime }\left(v,C|C_{1},\ldots ,C_{t}\right)$ such that the following holds:*

- *$W(v,C^{\prime })$ and $W(v,C_{i})$ are edge-disjoint for 1≤i≤t;*
- *$W\left(v^{\prime },C^{\prime }\right)=W\left(v^{\prime },C\right)$ for all $v^{\prime }\in V\setminus \left\{v\right\}$.*

Intuitively speaking, given the current Eulerian circuit *C* and previous circuits $C_{1},\ldots ,C_{t}$, we can rewire *v* with respect to *C* to obtain $C^{\prime }$ such that none of $C_{1},\ldots ,C_{t}$ uses the same in/out arc pair of *v* as $C^{\prime }$.

**Proof of Theorem 1.**  We first prove $\Omega_{\ell }\left(\sigma ,k\right)\geq \ell \left\lfloor \sigma /2\right\rfloor$ for σ≥4. Let $C_{1,1}$ be an Eulerian circuit of $G_{\sigma ,k}$. We arbitrarily partition the vertices in $G_{\sigma ,k}$ into *ℓ* groups, say $V_{1},\ldots ,V_{\ell }$. Then, for j∈[2,ℓ], we recursively define

(2) $$\begin{array}{c} C_{1,j}:=C^{\prime }\left(V_{j-1},C_{1,j-1}|C_{1,1}\right), \end{array}$$

where for a collection of vertices V the notation $C^{\prime }\left(V,C|C_{1},\ldots ,C_{t}\right)$ refers to an Eulerian circuit obtained by rewiring each vertex in V with respect to *C* given $C_{1},\ldots ,C_{t}$. Writing $V=\{v_{1},\ldots ,v_{q}\}$, we define $C^{\prime }\left(V,C|C_{1},\ldots ,C_{t}\right):=C^{\left(q\right)}$, where $C^{\left(0\right)}:=C$ and $C^{\left(q^{\prime }\right)}:=C^{\prime }\left(v_{q^{\prime }},C^{\left(q^{\prime }-1\right)}|C_{1},\ldots ,C_{t}\right),$ for $q^{\prime }\in \left[1,q\right]$.

**Remark 1.**  *It is noteworthy that a different labeling of elements in V may lead to different $C^{\prime }\left(V,C|C_{1},\ldots ,C_{t}\right)$. That is, $C^{\prime }\left(V,C|C_{1},\ldots ,C_{t}\right)$ is not unique. However, in this proof, we can use any one of $C^{\prime }\left(V,C|C_{1},\ldots ,C_{t}\right)$ to construct the circuits of interest.*

Next, write K⌊σ/2⌋. Note that every vertex in $G_{\sigma ,k}$ has in/out-degree σ. Thus, by Lemma 2, for any Eulerian circuit *C* and any vertex v of $G_{\sigma ,k}$, we can always rewire v with respect to *C* given any collection of less than *K* compatible Eulerian circuits. This ensures that the following recursive definitions hold: For i∈[2,K−1],

(3) $$\begin{array}{c} C_{i,1}:=C^{\prime }\left(V_{\ell },C_{i-1,\ell }|C_{1,1},\ldots ,C_{i-1,1}\right), \end{array}$$

and for j∈[2,ℓ],

(4) $$\begin{array}{c} C_{i,j}:=C^{\prime }\left(V_{j-1},C_{i,j-1}|C_{1,1},\ldots ,C_{i,1}\right). \end{array}$$

Next, define

(5) $$\begin{array}{c} C_{K,1}:=C^{\prime }\left(V_{\ell },C_{K-1,\ell }|C_{1,1},\ldots ,C_{K-1,1}\right), \end{array}$$

and for j∈[2,ℓ], let

(6) $$\begin{array}{c} C_{K,j}:=C^{\prime }\left(V_{j-1},C_{K,j-1}|C_{2,1},\ldots ,C_{K,1}\right). \end{array}$$

Note that in (6), the conditioned circuits start from $C_{2,1}$ instead of $C_{1,1}$. We claim that the following collection $C:=\{C_{i,j}\;|\;i\in \left[1,K\right],j\in \left[1,\ell \right]\}$ is *ℓ*-orthogonal. Let v be in the *m*th group of vertices, $V_{m},$ for some m∈[1,ℓ]. Observe that the following holds:

- For each i∈[1,K−1], the wiring of v is the same in all $C_{i,m+1},\ldots ,C_{i,\ell },C_{i+1,1},\ldots ,C_{i+1,m}$. Denote it by $W_{i}$. Denote by $W_{0}$ and $W_{K}$ the shared wiring of v in $C_{1,1},\ldots ,C_{1,m}$ and $C_{K,m+1},\ldots ,C_{K,\ell }$, respectively.
- The wirings $W_{i}$ and $W_{i^{\prime }}$ are edge-disjoint for i∈[0,K], $i^{\prime }\in \left[1,K-1\right]$ and $i\neq i^{\prime }$. Thus, each in/out arc pair of v used in any $W_{1},\ldots ,W_{K-1}$ is used exactly *ℓ* times in C.
- Even if $W_{0}$ and $W_{K}$ share some edge in their bipartite graphs, that edge is used only *ℓ* times (*m* times by $W_{0}$ and ℓ−m times by $W_{K}$). This establishes the claim.

Now we prove $\Omega_{\ell }\left(\sigma ,k\right)\geq 2\ell$ as long as σ≥3. Again, let $C_{1,1}$ be an Eulerian circuit of $G_{\sigma ,k}$. We recursively apply Lemma 1 to define for j∈[2,ℓ] that

(7) $$\begin{array}{c} C_{1,j}:=C^{\prime }\left(V_{j-1},C_{1,j-1}\right), \end{array}$$

Next, define

(8) $$\begin{array}{c} C_{2,1}:=C^{\prime }\left(V_{\ell },C_{1,\ell }\right), \end{array}$$

and for j∈[2,ℓ], let

(9) $$\begin{array}{c} C_{2,j}:=C^{\prime }\left(V_{j-1},C_{2,j-1}\right). \end{array}$$

A similar argument shows that $C:=\{C_{i,j}\;|\;i\in \left\{1,2\right\},j\in \left[1,\ell \right]\}$ is *ℓ*-orthogonal. □

**Figure 2:**
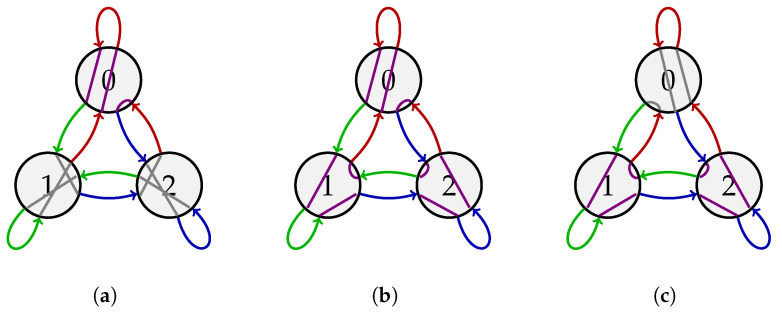
(**a**) The circuit $C_{1,2}=012022110$ obtained by rewiring $C_{1,1}=012002211$ at the vertex 0. (**b**) The circuit $C_{2,1}=011220210$ obtained by rewiring $C_{1,2}$ at the vertices 1 and 2. (**c**) The circuit $C_{2,2}=011220021$ obtained by rewiring $C_{2,1}$ at the vertex 0.

**Example 2.**  *This example demonstrates the rewiring process from the proof of Theorem 1. Consider k=2, A={0,1,2}, and ℓ=2. We seek to find 2ℓ=4 Eulerian circuits on $G_{3,2}$ such that each in/out arc pair of each vertex is used at most twice. We first partition $V\left(G_{3,2}\right)=\left\{0,1,2\right\}$ into $V_{1}=\left\{0\right\}$ and $V_{2}=\left\{1,2\right\}$. Also, we select the starting circuit $C_{1,1}$ to be the Eulerian circuit in $G_{3,2}$ for the (3,2)-de Bruijn sequence *012002211*. With a slight abuse of notation, we write $C_{1,1}=012002211$. According to *(7)–(9)*, the rewired circuits are $C_{1,2}=012022110$, $C_{2,1}=011220210$, and $C_{2,2}=011220021$ (Figure 2). Comparing Figure 1b and Figure 2, we see that each vertex has two edge-disjoint wirings (gray and violet), each of which appears twice in the collection $\left\{C_{1,1},C_{1,2},C_{2,1},C_{2,2}\right\}$.*

## 4. Orthogonal Balanced de Bruijn Sequences

We start with several definitions relevant to the balanced de Bruijn sequence defined in Definition 3.

**Definition 6.**  *A b-balanced (σ,k)-de Bruijn sequence s is* self-orthogonal *if any sequence in $A^{k+1}$ appears at most once in s. A collection of b-balanced (σ,k)-de Bruijn sequences $C=\{s_{1},\ldots ,s_{M}\}$ is called* orthogonal *if each sequence in $A^{k+1}$ appears at most once in C.*

**Remark 2.**  *Clearly, a b-balanced de Bruijn sequence in an orthogonal collection is self-orthogonal.*

**Definition 7.**  *Let b≥1, G be a directed graph, and let C be a circuit of G. We say that C is ab*-circuit *of G if C visits each vertex of G exactly b times.*

**Remark 3.**  *A b-circuit is sometimes referred to as an exact b-walk [13].*

**Proposition 2.**  *There is a one-to-one correspondence between self-orthogonal b-balanced (σ,k)-de Bruijn sequences and b-circuits on $G_{\sigma ,k+1}$. A collection of b-balanced (σ,k)-de Bruijn sequences is orthogonal if and only if each sequence is self-orthogonal and their corresponding b-circuits are arc-disjoint.*

**Figure 3:**
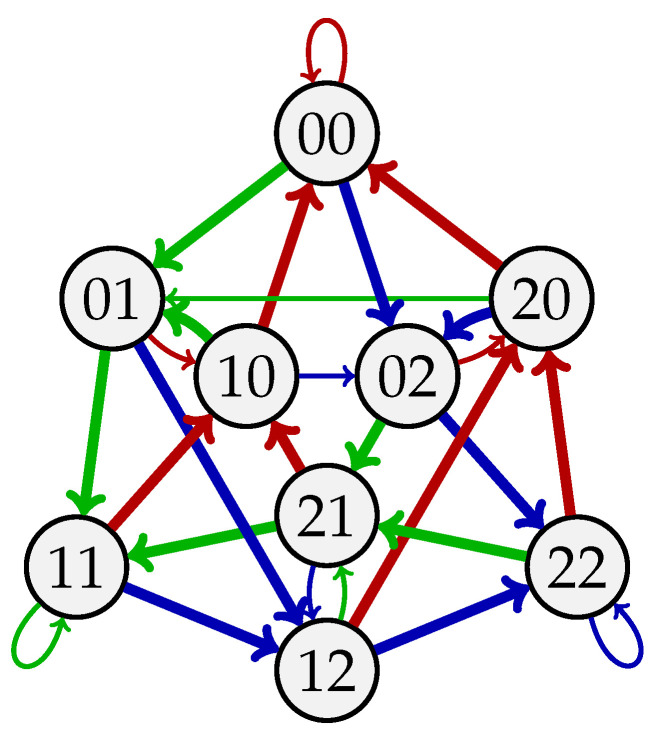
The de Bruijn graph $G_{3,3}$. The bold arcs correspond to the 2-circuit representing the 2-balanced (3,2)-de Bruijn sequence 002211012001122021.

**Example 3.**  *Consider k=2, A={0,1,2}, and b=2. The circular sequences $s_{1}=000111222020212101$ and $s_{2}=002211012001122021$ are two 2-balanced (3,2)-de Bruijn sequences since each length-2 sequence over {0,1,2} appears exactly twice in both $s_{1}$ and $s_{2}$. The sequence $s_{1}$ is not self-orthogonal since the length-3 sequence 202 appears twice in $s_{1}$. On the other hand, $s_{2}$ is self-orthogonal, and it corresponds to the 2-circuit 00→02→22→21→11→10→01→12→20→00→01→11→12→22→20→02→21→10→00 on $G_{3,3}$ (see Figure 3).*

Fix σ. Observe that when *b* increases, the number of length-(k+1) sequences in any two *b*-balanced (σ,k)-de Bruijn sequences increases as well, making them less likely to be orthogonal. This motivates the following definition: For c≥2, define Σ(c,b,k) to be the minimum σ such that there exist *c* orthogonal *b*-balanced (σ,k)-de Bruijn sequences.

We start by establishing a lower bound on Σ(c,b,k).

**Proposition 3.**  *If b≥2, then Σ(c,b,k)≥cb.*

**Proof.**  Similar to the proof of Proposition 1, we observe that there are σ outgoing arcs for each vertex in $G_{\sigma ,k+1}$. By Proposition 2, a self-orthogonal *b*-balanced de Bruijn sequence uses exactly *b* incoming and outgoing arcs at each vertex. Therefore, since *c* orthogonal *b*-balanced de Bruijn sequences share no arcs, they must use cb distinct outgoing arcs at each vertex. This is impossible if σ<cb. □

We also have the following upper bound on Σ(c,b,k).

**Theorem 2.**  *For b≥2, Σ(c,b,k) is at most the smallest prime power that is greater than or equal to cb. Furthermore, Σ(c,b,k)=cb if each prime factor of c divides b.*

To prove Theorem 2, we establish the following lemmas.

**Lemma 3.**  *Assume b≥2, $\sigma =p^{m}$ for some prime p and positive integer m, and σ≥cb. Then there exist c orthogonal b-balanced (σ,k)-de Bruijn sequences.*

**Proof.**  Since σ is a prime power, by [10] Lemma 2, there exist cycles $C_{0},\ldots ,C_{\sigma -1}$ on $G_{\sigma ,k+1}$ such that the following holds:

- For i,j∈[0,σ−1] with i≠j, the cycles $C_{i}$ and $C_{j}$ are arc-disjoint.
- For i∈[0,σ−1], the cycle $C_{i}$ does not visit the vertex representing the sequence ii⋯i (assuming A=[0,σ−1]) and visits all the other vertices exactly once.

Then, for each t∈[0,c−1], we can construct a circuit ${\hat{C}}_{t}$ as follows. Let ${\tilde{C}}_{bt+i}$ be the cycle $C_{bt+i}$ with an additional transition following the loop on the vertex representing the all-(bt+i+1) sequence, where i∈[0,b−2]. Note that this definition is valid since $C_{bt+i}$ must visit the all-(bt+i+1) sequence. Also, let ${\tilde{C}}_{bt+b-1}$ be the cycle $C_{bt+b-1}$ with an additional transition following the loop at the all-(bt) vertex. Then, let ${\hat{C}}_{t}$ be a combination of ${\tilde{C}}_{bt},\ldots ,{\tilde{C}}_{bt+b-1}$ (a combination of ${\tilde{C}}_{bt},\ldots ,{\tilde{C}}_{bt+b-1}$ is a closed walk whose arcs are exactly those in ${\tilde{C}}_{bt},\ldots ,{\tilde{C}}_{bt+b-1}$). Since each pair of ${\tilde{C}}_{i},{\tilde{C}}_{j}$ meets at some vertex, such a closed walk always exists. Then, observe the following: (a) the arcs used in ${\hat{C}}_{t}$ are those in $C_{bt},\ldots ,C_{bt+b-1}$ and the loops at the all-(bt+i) vertices for i∈[0,b−1]; (b) $C_{bt},\ldots ,C_{bt+b-1}$ are arc-disjoint and do not use any loops; (c) for i∈[0,b−1], the all-(bt+i) vertex is visited *b* times in ${\hat{C}}_{t}$ (twice by ${\hat{C}}_{bt+((i-1)\;\mathrm{mod}\;b)}$, never by ${\hat{C}}_{bt+i}$, and once by each remaining ${\hat{C}}_{bt+j}$); (d) each of the remaining vertices in $G_{\sigma ,k+1}$ is also visited *b* times in ${\hat{C}}_{t}$ (once by each ${\hat{C}}_{bt+j}$). Therefore, ${\hat{C}}_{t}$ is a *b*-circuit on $G_{\sigma ,k+1}$. The arc-disjointness and no-loop property of the $C_{i}$s imply that ${\hat{C}}_{t}$ and ${\hat{C}}_{t^{\prime }}$ are arc-disjoint for $t\neq t^{\prime }$. Thus, ${\hat{C}}_{0},\ldots ,{\hat{C}}_{c-1}$ are orthogonal *b*-balanced (σ,k)-de Bruijn sequences. □

**Lemma 4.**  *Let $G_{1}=\left(V_{1},A_{1}\right)$ and $G_{2}=\left(V_{2},A_{2}\right)$ be two directed graphs. Write $N_{1}:=\left|V_{1}\right|$ and $N_{2}:=\left|V_{2}\right|$. Assume $G_{1}$ has $n_{1}$ arc-disjoint $b_{1}$-circuits and $G_{2}$ has $n_{2}$ arc-disjoint $b_{2}$-circuits. If $N_{1}b_{1}$ and $N_{2}b_{2}$ are coprime, then the tensor-product graph $G_{1}\times G_{2}$ has $n_{1}n_{2}$ arc-disjoint $b_{1}b_{2}$-circuits.*

**Proof.**  Let $C_{1}^{\left(1\right)},\ldots ,C_{1}^{\left(n_{1}\right)}$ be arc-disjoint $b_{1}$-circuits of $G_{1}$ and $C_{2}^{\left(1\right)},\ldots ,C_{2}^{\left(n_{2}\right)}$ be arc-disjoint $b_{2}$-circuits of $G_{2}$.

Then, for each $i\in [1,n_{1}]$ and $j\in [1,n_{2}]$, define $C_{i,j}$ to be the subgraph of $G_{1}\times G_{2}$ with arc set

$$\begin{array}{cc} A_{i,j} & :=\left\{\left(\left(v_{1},v_{2}\right),\left(v_{1}^{\prime },v_{2}^{\prime }\right)\right)\;|\;\left(v_{1},v_{1}^{\prime }\right)\in A\left(C_{1}^{\left(i\right)}\right)\;\mathrm{and}\;\left(v_{2},v_{2}^{\prime }\right)\in A\left(C_{2}^{\left(j\right)}\right)\right\}, \end{array}$$

where for a directed graph *G*, we use A(G) to denote its arc set.

We first show that $C_{i,j}$ is a circuit. Since $C_{1}^{\left(i\right)}$ is a circuit of length $N_{1}b_{1}$, we can traverse $C_{1}^{\left(i\right)}$ following a circular sequence of distinct arcs $\left(a_{1},a_{2},\ldots ,a_{N_{1}b_{1}}\right)$, where each $a_{k}\in A_{1}$ and the target of $a_{k}$ equals the source of $a_{k+1}$. Similarly, we can represent $C_{2}^{\left(j\right)}$ by a circular sequence of arcs $\left(d_{1},d_{2},\ldots ,d_{N_{2}b_{2}}\right)$, where each $d_{\ell }\in A_{2}$ and the target of $d_{k}$ equals the source of $d_{k+1}$. Then, with a slight abuse of notation, we write $A_{i,j}=\left\{\left(a_{k},d_{\ell }\right)\;|\;k\in [1,N_{1}b_{1}],\ell \in [1,N_{2}b_{2}]\right\}.$ Now fix an arc $\left(a_{k},d_{\ell }\right)$ of $C_{i,j}$. We can traverse $C_{i,j}$ starting from $\left(a_{k},d_{\ell }\right)$ and by following $\left(a_{k+1},d_{\ell +1}\right)$, $\left(a_{k+2},d_{\ell +2}\right)$, etc. Then, the next time $\left(a_{k},d_{\ell }\right)$ is used is after $\mathrm{lcm}\left(N_{1}b_{1},N_{2}b_{2}\right)=N_{1}N_{2}b_{1}b_{2}$ steps. However, note that there are exactly $N_{1}N_{2}b_{1}b_{2}$ arcs in $C_{i,j}$. Therefore, right before the next use of $\left(a_{k},d_{\ell }\right)$ in $C_{i,j}$, each arc in $C_{i,j}$ has been traversed, and thus $C_{i,j}$ represents a single closed walk on $G_{1}\times G_{2}$. Furthermore, if some arc is repeated, starting the traversal from that arc would contradict the fact that the next use of each arc has to happen after $N_{1}N_{2}b_{1}b_{2}$ steps. Hence, $C_{i,j}$ is a circuit. Then, since $C_{1}^{\left(i\right)}$ and $C_{1}^{\left(i^{\prime }\right)}$ are arc-disjoint for $i\neq i^{\prime }$ and $C_{2}^{\left(j\right)}$ and $C_{2}^{\left(j^{\prime }\right)}$ for $j\neq j^{\prime }$, we have that $C_{i,j}$ and $C_{i^{\prime },j^{\prime }}$ are arc-disjoint when either $i\neq i^{\prime }$ or $j\neq j^{\prime }$.

To verify that each $C_{i,j}$ is a $b_{1}b_{2}$ circuit, it remains to show that each $C_{i,j}$ visits each vertex in $G_{1}\times G_{2}$ exactly $b_{1}b_{2}$ times. Fix a vertex *v* in $G_{1}\times G_{2}$. Write $v=(v_{1},v_{2})$ for some $v_{1}\in V_{1}$ and $v_{2}\in V_{2}$. On $C_{1}^{\left(i\right)}$, the vertex $v_{1}$ has exactly $b_{1}$ outgoing arcs, say $a_{1}^{\prime },\ldots ,a_{b_{1}}^{\prime }$. Similarly, the outgoing arcs of the vertex $v_{2}$ on $C_{2}^{\left(j\right)}$ are denoted by $d_{1}^{\prime },\ldots ,d_{b_{2}}^{\prime }$. Therefore, the outgoing arcs of $\left(v_{1},v_{2}\right)$ on $C_{i,j}$ are of the form $\left(a_{k^{\prime }}^{\prime },d_{\ell^{\prime }}^{\prime }\right)$, where $k^{\prime }\in \left[1,b_{1}\right]$ and $\ell^{\prime }\in \left[1,b_{2}\right]$. Since $\left(v_{1},v_{2}\right)$ has exactly $b_{1}b_{2}$ outgoing arcs in $C_{i,j}$, it is visited exactly $b_{1}b_{2}$ times in $C_{i,j}$. Since *v* is arbitrary, $C_{i,j}$ is a $b_{1}b_{2}$-circuit.

In conclusion, we have shown that ${\left\{C_{i,j}\right\}}_{i\in [1,n_{1}],j\in [1,n_{2}]}$ is a collection of $n_{1}n_{2}$ arc-disjoint $b_{1}b_{2}$-circuits. □

**Lemma 5.**  *The tensor-product graph $G_{\sigma_{1},k}\times G_{\sigma_{2},k}$ is isomorphic to $G_{\sigma_{1}\sigma_{2},k}$.*

**Sketch of the proof.**  Without loss of generality, assume that the alphabets of $G_{\sigma_{1},k}$, $G_{\sigma_{2},k}$, and $G_{\sigma_{1}\sigma_{2},k}$ are $\left[0,\sigma_{1}-1\right]$, $\left[0,\sigma_{2}-1\right]$, and $\left[0,\sigma_{1}\sigma_{2}-1\right]$, respectively. It is not hard to show that the following mapping is a graph isomorphism:

(10) $$\begin{array}{cc} G_{\sigma_{1}\sigma_{2},k} & \to G_{\sigma_{1},k}\times G_{\sigma_{2},k}\\ \left(s_{0},\ldots ,s_{k-2}\right) & \mapsto (\left(q_{0},\ldots ,q_{k-2}\right),\left(r_{0},\ldots ,r_{k-2}\right)), \end{array}$$

where for i∈[0,k−2], we write $q_{i}$ and $r_{i}$ for the quotient and remainder of $s_{i}$ when divided by $\sigma_{2}$, respectively. Note that the construction in (10) is related to the proofs of [10] Lemma 3 and [14] Lemma 3. □

**Sketch of the proof of Theorem 2.**  Lemma 3 established the first statement in the theorem. Now, assume that each prime factor of *c* divides *b*. Then, we can write $c=p_{1}^{x_{1}}\cdots p_{m}^{x_{m}}$ and $b=p_{1}^{y_{1}}\cdots p_{m}^{y_{m}}R$, where $p_{1},\ldots ,p_{m}$ are distinct prime numbers, $x_{1},\ldots ,x_{m},y_{1},\ldots ,y_{m}$ are positive integers, and none of the $p_{1},\ldots ,p_{m}$ divides *R*.

Now consider the following m+1 de Bruijn graphs $G_{p_{1}^{x_{1}+y_{1}},k+1},\ldots ,G_{p_{m}^{x_{m}+y_{m}},k+1},$ $G_{R,k+1}$. By Lemma 3, for each i∈[1,m], $G_{p_{i}^{x_{i}+y_{i}},k+1}$ has $p_{i}^{x_{i}}$ arc-disjoint $p_{i}^{y_{i}}$-circuits. We also know that $G_{R,k+1}$ has one *R*-circuit, which is essentially an Eulerian circuit on it. Then, by repeatedly applying Lemma 4 and Lemma 5, we can see that $G_{\prod_{i=1}^{m}p_{i}^{x_{i}+y_{i}}R,k+1}$ has $\prod_{i=1}^{m}p_{i}^{x_{i}}$ arc-disjoint $\left(\prod_{i=1}^{m}p_{i}^{y_{i}}R\right)$-circuits. Equivalently, $G_{cb,k+1}$ has *c* arc-disjoint *b*-circuits. Therefore, Σ(c,b,k)≤cb. We conclude that Σ(c,b,k)=cb by invoking Proposition 3. □

**Example 4.**  *Consider c=2, b=6, and k=2 in Theorem 2. In this case, $p_{1}=2$, $x_{1}=y_{1}=1$, and R=3. Next, examine the two de Bruijn graphs $G_{4,3}$ and $G_{3,3}$. We associate $G_{3,3}$ with the Eulerian circuit E=100020212210222001012112011.*

*Lemma 3 implies that $G_{4,3}$ has two arc-disjoint 2-circuits, constructed as follows. The cycles $C_{0},C_{1},C_{2},C_{3}$ are*

(11) $$\begin{array}{c} C_{0}=011310221203323,\;C_{1}=100201330312232,\\ C_{2}=233132003021101,\;C_{3}=322023112130010. \end{array}$$

*Then, the circuit ${\tilde{C}}_{0}$ is defined by $C_{0}$ following the loop on the vertex *11*. Written as a sequence, ${\tilde{C}}_{0}=0111310221203323$. Note that the loop on *11 *induces the length*-3 *substring *111 *in ${\tilde{C}}_{0}$. Similarly, we have*

(12) $$\begin{array}{c} {\tilde{C}}_{1}=1000201330312232,\;{\tilde{C}}_{2}=2333132003021101,\\ {\tilde{C}}_{3}=3222023112130010. \end{array}$$

*Let ${\hat{C}}_{0}$ and ${\hat{C}}_{1}$ be a combination of ${\tilde{C}}_{0}$ and ${\tilde{C}}_{1}$ and one of ${\tilde{C}}_{2}$ and ${\tilde{C}}_{3}$, respectively. For example, we can take*

$$\begin{array}{c} {\hat{C}}_{0}=01113102212033230133031223210002,\\ {\hat{C}}_{1}=23331320030211012311213001032220. \end{array}$$

*Then, ${\hat{C}}_{0}$ and ${\hat{C}}_{1}$ are arc-disjoint *2*-circuits on $G_{4,3}$.*

*Then, we define $A_{0}=3{\hat{C}}_{0}+E$ and $A_{1}=3{\hat{C}}_{1}+E$, where we treat ${\hat{C}}_{0}$, ${\hat{C}}_{1}$, and E as infinitely long periodic sequences, and with additions performed entry-wise. Here the coefficient 3 in front of ${\hat{C}}_{0}$ and ${\hat{C}}_{1}$ arises from the size of the alphabet of E. The first few terms of $A_{0}$ are (1,3,3,3,11,…), and the period of $A_{0}$ is lcm(32,27)=864 since ${\hat{C}}_{0}$ has period *32 *and E has period *27*. By Lemmas 4 and 5, $A_{0}$ and $A_{1}$ correspond to two arc-disjoint *6*-circuits on $G_{12,3}$.*

## 5. Orthogonal Fixed-Weight de Bruijn Sequences

As pointed out, in many applications we are allowed to only use sequences with constrained compositions. We therefore first generalize the definition of de Bruijn sequences in a way that its length-*k* substrings belong to a constrained subset of length-*k* sequences.

**Definition 8**  ([6] Section 1)**.** *Let k≥1, A be an alphabet of size σ≥2, and let L be a subset of $A^{k}$. A circular sequence $s=(s_{0},s_{1},\ldots ,s_{|L|-1})$ is called a* de Bruijn sequence with respect to *L if each sequence in L appears as a circular substring of s exactly once.*

Similar to the nonrestricted case, restricted de Bruijn sequences can be characterized as Eulerian circuits of a specialized directed graph, defined as follows.

**Definition 9**  ([6] Section 2; [4] Section 3)**.** *Let k≥1, and let A be an alphabet of size σ≥2. Let L be a proper subset of $A^{k}$. Define the* de Bruijn graph with respect to *L as a directed graph D(L) with the vertex set V(D(L)) being the collection of all length-(k−1) prefixes and length-(k−1) suffixes of sequences in L, and with arc set*

(13) $$\begin{array}{cc} A(D(L))\; & :=\{\left(s_{1},s_{2}\right)\in V{\left(D\left(L\right)\right)}^{2}\;|\;\mathrm{there}\;\mathrm{is}\;a\;\mathrm{sequence}\;\mathrm{in}\;L\;\mathrm{with}\;\mathrm{prefix}\;s_{1}\;\mathrm{and}\;\mathrm{suffix}\;s_{2}\}. \end{array}$$

Then, there is a one-to-one correspondence between de Bruijn sequences with respect to *L* and Eulerian circuits of D(L). Note that for some choices of *L*, there may not exist a de Bruijn sequence with respect to *L* or an Eulerian circuit in D(L).

We say that two de Bruijn sequences with respect to *L* are *orthogonal* if they have no common length-(k+1) circular substring. Similarly, two de Bruijn sequences with respect to *L* are orthogonal if and only if their corresponding Eulerian circuits on D(L) are compatible. We seek to analyze the restricted de Bruijn graph D(L) in Definition 8, where *L* is the collection of all sequences of certain restricted weights.

Formally, write A=W∪X, where W represents the set of “weighted symbols”, while X represents the set of “nonweighted” symbols, with both W and X nonempty and disjoint. Then, for any n≥1 and each $s=\left(s_{0},\ldots ,s_{n-1}\right)\in A^{n}$, we define the weight of s, ω(s), as the number of entries of s in W: $\omega \left(s\right):=\sum_{i=0}^{n-1}{𝟙}_{\{s_{i}\in W\}}.$

Then, for $0\leq w^{\prime }\leq w\leq k,$ we define $A_{w^{\prime }}^{w}\left(k\right)$ to be the collection of all sequences in $A^{k}$ with weight between $w^{\prime }$ and *w*. Our interest lies in orthogonal de Bruijn sequences with respect to $A_{w-1}^{w}\left(k\right)$ for w∈[1,k], i.e., compatible Eulerian circuits on $D(A_{w^{\prime }}^{w}\left(k\right))$ when *w* and $w^{\prime }$ differ by 1. Note that this is the smallest difference between *w* and $w^{\prime }$ we can consider due to the following fact: If $w^{\prime }=w$, then $D\left(A_{w^{\prime }}^{w}\left(k\right)\right)=D\left(A_{w}^{w}\left(k\right)\right)$ is not strongly connected and thus not Eulerian unless w∈{0,1,k−1,k} [6]. The reason is that any vertex whose weight representation contains the substring 1010 can never reach a vertex with weight representation 111⋯000. Here, the weight representation of $s=(s_{0},\ldots ,s_{k-2})$, denoted by χ(s), is the binary vector $\chi \left(s\right):=\left({𝟙}_{s_{0}\in W},\ldots ,{𝟙}_{s_{k-2}\in W}\right).$

Next, we generalize the arguments in [6] and characterize the vertices of $D(A_{w-1}^{w}\left(k\right)),$ as well as their degrees. To simplify our discussion, we further assume that w∈[2,k−1]. First, note that any length-(k−1) prefix or suffix of any string from $A_{w-1}^{w}\left(k\right)$ is necessarily of weight w−2, w−1, or *w*. Conversely, any length-(k−1) string whose weight is between w−2 and *w* can be seen as a prefix of some string in $A_{w-1}^{w}\left(k\right)$. As a result, the vertex set $V(D\left(A_{w-1}^{w}\left(k\right)\right))$ is equal to the set $A_{w-2}^{w}\left(k-1\right)$. Then, for $s=\left(s_{0},\ldots ,s_{k-2}\right)\in V\left(D\left(A_{w-1}^{w}\left(k\right)\right)\right)$ with ω(s)=w−2, we observe that its predecessors and successors are $\left\{\left(\alpha ,s_{0},\ldots ,s_{k-3}\right)|\alpha \in W\right\}$ and $\left\{\left(s_{1},\ldots ,s_{k-2},\beta \right)|\beta \in W\right\}$, respectively. Thus, the in-degree and out-degree of each $s\in V(D\left(A_{w-1}^{w}\left(k\right)\right))$ with ω(s)=w−2 are equal to |W|. Similarly, for $s\in V(D\left(A_{w-1}^{w}\left(k\right)\right))$ with ω(s)=w, the in- and out-degrees equal |X|. For $s\in V(D\left(A_{w-1}^{w}\left(k\right)\right))$ with ω(s)=w−1, there are no restrictions, and thus, its in- and out-degrees equal |A|=|W|+|X|.

The following theorem characterizes the number of orthogonal de Bruijn sequences with respect to $A_{w-1}^{w}\left(k\right)$:

**Theorem 3.**  *Let w∈[2,k−1]. The de Bruijn graph $D(A_{w-1}^{w}\left(k\right))$ has a collection of min(|W|,|X|) compatible Eulerian circuits. Furthermore, this is the largest possible collection. In particular, $A_{w-1}^{w}\left(k\right)$ has an Eulerian circuit.*

This finding is consistent with the results of [4] Proposition 3, which state that $D(A_{w^{\prime }}^{w}\left(k\right))$ always has an Eulerian circuit as long as $w^{\prime }<w$.

**Proof of Theorem 3.**  Without loss of generality, we assume that |W|≤|X| since the other case can be handled similarly. Since each vertex in the de Bruijn graph $D(A_{w-1}^{w}\left(k\right))$ has in/out-degree |W|, |X|, or |W|+|X|, there is no collection of more than |W| pairwise compatible Eulerian circuits of $D(A_{w-1}^{w}\left(k\right))$.

Before we proceed, we recall the vertex-splitting technique mentioned in [9]: Given a directed graph G=(V,A), a vertex *v* with both in-degree and out-degree *d* that has no loop, and a wiring (transition system) *W* on *v*, we can define another directed graph $G^{\prime }=\left(V^{\prime },A^{\prime }\right)$ by “splitting *v* along *W*” as follows: The vertex set of $G^{\prime }$ is $V^{\prime }=V\cup \left\{v_{1},v_{2},\ldots ,v_{d}\right\}\setminus \left\{v\right\}$, where $v_{1},\ldots ,v_{d}$ are newly introduced vertices. In other words, we remove *v* from *G* and add *d* new vertices. Each $v_{j}$ has in/out-degree 1, and its predecessor and successor are determined by exactly one edge of *W*. The arcs on all the other vertices V∖{v} remain the same as in *G*.

Then, we enumerate $W=\{w_{0},w_{1},\ldots ,w_{|W|-1}\}$ and $X=\{x_{0},x_{1},\ldots ,x_{|X|-1}\}$. For each vertex $s=(s_{0},\ldots ,s_{k-2})$ of $D(A_{w-1}^{w})$ of weight w−2, we can define |W| edge-disjoint wirings $W_{0}\left(s\right),\ldots ,W_{|W|-1}\left(s\right)$ on s by the following rule: For j∈[0,|W|−1], the wiring $W_{j}\left(s\right)$ is defined by pairing the incoming arc $\left(w_{i},s_{0},\ldots ,s_{k-3}\right)$ with the outgoing arc $\left(s_{1},\ldots ,s_{k-2},w_{\left(i+j)\;\mathrm{mod}\;|W\right|}\right)$ for each i∈[0,|W|−1]. Similarly, for each vertex $v=(v_{0},\ldots ,v_{k-2})$ in $D(A_{w-1}^{w})$ of weight *w* and for j∈[0,|W|−1], we define the wiring $W_{j}^{\prime }\left(v\right)$ by pairing the incoming arc $\left(x_{i},s_{0},\ldots ,s_{k-3}\right)$ with the outgoing arc $\left(s_{1},\ldots ,s_{k-2},x_{\left(i+j)\;\mathrm{mod}\;|X\right|}\right)$ for each i∈[0,|X|−1]. Recall that |W|≤|X|, and thus $W_{0}^{\prime }\left(v\right),\ldots ,W_{|W|-1}^{\prime }\left(v\right)$ are edge-disjoint as well.

Now, for each j∈[0,|W|−1], we define $G_{j}$ to be the directed graph obtained from $D(A_{w-1}^{w}\left(k\right))$ by splitting *every* vertex s of weight w−2 along $W_{j}\left(s\right)$ and splitting *every* vertex v of weight *w* along $W_{j}^{\prime }\left(v\right)$. If each $G_{j}$ admits an Eulerian cycle, then by “merging back the splitted vertices”, we obtain |W| Eulerian circuits $C_{0},\ldots ,C_{|W|-1}$ on $D(A_{w-1}^{w}\left(k\right))$ such that for each vertex with weight w−2 or *w*, no in/out arc-pair is used twice in these $C_{j}$. Then, since the remaining weight-(w−1) vertices have degree |W|+|X|≥2|W|, for each *j* we can repeatedly apply Lemma 2 to rewire each weight-(w−1) vertex in $C_{j}$ given $C_{0},\ldots ,C_{j-1}$. After this process, we obtain |W| compatible Eulerian circuits on $D(A_{w-1}^{w}\left(k\right))$.

It remains to show that each $G_{j}$ indeed has an Eulerian circuit. Since each vertex in $G_{j}$ either has in/out degree 1 or in/out degree |W|+|X|, it suffices to verify that $G_{j}$ is strongly connected. Let $v=(v_{0},\ldots ,v_{k-2})$ and $v^{\prime }=\left(v_{0}^{\prime },\ldots ,v_{k-2}^{\prime }\right)$ be two vertices in $G_{j}$. We first show that from v, we can reach $v^{\prime }$ if $v^{\prime }$ has weight w−1. Write the weight representations of v and $v^{\prime }$ as $\chi \left(v\right)=\left(b_{0},\ldots ,b_{k-2}\right)$ and $\chi \left(v^{\prime }\right)=\left(b_{0}^{\prime },\ldots ,b_{k-2}^{\prime }\right)$, respectively. Also, consider the following generalized de Bruijn graph $D(B_{w-1}^{w}\left(k\right))$, where B consists of only one weighted symbol 1 and one nonweighted symbol 0. By [6] Corollary 2.3, there is a path *P* from χ(v) to $\chi (v^{\prime })$ on $D(B_{w-1}^{w}\left(k\right))$. Write the binary sequence induced by *P* as $p=(a_{0},\ldots a_{T})$, where $\left(a_{0},\ldots ,a_{k-2}\right)=\left(b_{0},\ldots ,b_{k-2}\right)$ and $\left(a_{T-k+2},\ldots ,a_{T}\right)=(b_{0}^{\prime },\ldots ,b_{k-2}^{\prime })$. Then, observe that we can “follow the same path *P*” to walk on $G_{j}$ from v to some vertex having the same weight representation as $v^{\prime }$. That is, there is a path $P^{\prime }$ on $G_{j}$ from v to some $v^{\prime \prime }=\left(v_{0}^{\prime \prime },\ldots ,v_{k-2}^{\prime \prime }\right)$ such that the induced sequence of $P^{\prime }$ has weight representation p and the weight representation of $v^{\prime \prime }$ satisfies $\chi \left(v^{\prime \prime }\right)=\chi \left(v^{\prime }\right)$. This claim can be easily established by induction on the length of *P*.

Then, note that since $v^{\prime \prime }$ and $v^{\prime }$ have the same weight representation and they all share the same weight w−1, we can traverse from $v^{\prime \prime }$ to $v^{\prime }$ using a walk $P^{\prime \prime }$ that induces the sequence $p^{\prime \prime }=\left(v_{0}^{\prime \prime },\ldots ,v_{k-2}^{\prime \prime },v_{0}^{\prime },\ldots ,v_{k-2}^{\prime }\right)$. The weight representation of each length-*k* substring in $p^{\prime \prime }$ has a prefix that is a circular shift of $\chi (v^{\prime })$, and thus each length-*k* substring in $p^{\prime \prime }$ has weight *w* or w−1. Therefore, $P^{\prime \prime }$ is a valid walk in $D(A_{w-1}^{w}\left(k\right))$. Furthermore, each length-(k−1) substring in $p^{\prime \prime }$ has a weight representation equal to a circular shift of $\chi (v^{\prime })$, and thus the walk $P^{\prime \prime }$ in $D(A_{w-1}^{w}\left(k\right))$ only visits vertices of weight w−1. Thus, the walk $P^{\prime \prime }$ is unaffected by the splitting process and remains present in $G_{j}$. In conclusion, we can walk on $G_{j}$ from v to $v^{\prime \prime }$ and then from $v^{\prime \prime }$ to $v^{\prime }$, provided that $v^{\prime }$ has weight w−1.

Now consider the case where $v^{\prime }$ has weight *w* or w−2. If $v^{\prime }$ has weight w−2, we can traverse in a reverse direction from $v^{\prime }$ to its unique predecessor $\mathrm{pred}(v^{\prime })$, and then if $\mathrm{pred}(v^{\prime })$ still has weight w−2, traverse to $\mathrm{pred}(\mathrm{pred}\left(v^{\prime }\right))$, and so on. Since each traversal appends a symbol of weight 1 to the left of $v^{\prime }$ and removes the right-most symbol of $v^{\prime }$, we will reach a weight-(w−1) vertex (say $\hat{v}$) in $G_{j}$ after k−t+1 reverse steps, where $b_{t}^{\prime }$ is the last 0 in $\chi (v^{\prime })$. We can then apply the previous result to see that we can start from v to reach $\hat{v}$, and then, since $\hat{v}$ is obtained from a reverse traversal from $v^{\prime }$, walk from $\hat{v}$ to $v^{\prime }$. A similar argument holds when $v^{\prime }$ has weight *w*.

These arguments show that $G_{j}$ is strongly connected and thus has an Eulerian circuit. □

**Example 5.**  *Consider W={C,G}, X={A,T}, k=4, and w=3. Then, A={A,T,C,G} and $A_{w-1}^{w}\left(k\right)=A_{2}^{3}\left(4\right)$ represent the collection of all length*-4 *sequences over {A,T,C,G} with weight *2 *or* 3*, where the “weight” of a word is determined by the number of symbols that are either C or G (i.e., the GC content). The vertices of the de Bruijn graph $D(A_{2}^{3}\left(4\right))$ are all length*-3 *sequences of weight *1*, *2*, or *3*. For example, consider the vertex CAA, which has weight *1*. It has two incoming arcs, one from CCA and the other from GCA. The two outgoing arcs of this vertex point toward AAC and AAG, respectively.*

*For each vertex $\tilde{v}$ of $D(A_{2}^{3}\left(4\right))$ with weight *1 *or *3*, the wiring $W_{0}\left(\tilde{v}\right)$ associates each of its predecessors p to the successor s such that the first symbol of p equals the last symbol of s. For example, the vertex $\hat{v}=CAA$ has predecessors $p_{0}=CCA,p_{1}=GCA$ and successors $s_{0}=AAC,s_{1}=AAG$, and the wiring $W_{0}\left(\hat{v}\right)$ pairs $p_{0}$ with $s_{0}$ and $p_{1}$ with $s_{1}$. Then, in the split graph $G_{0}$, the original vertex $\hat{v}=CAA$ is split to two vertices $CAA_{C}$ and $CAA_{G}$, where the subscript C or G denotes the first symbol of its only predecessor, which is equal to the last symbol of its only successor. More explicitly, $CAA_{C}$ has only one incoming arc from CCA and only one outgoing arc toward $AAC_{C}$, and $CAA_{G}$ has only one incoming arc from GCA and only one outgoing arc toward $AAG_{C}$.*

We show next that from $v=CAA_{G}$, we can, for example, reach $v^{*}=TCT_{G}$ on this split graph $G_{0}$. First, since $v^{*}=TCT_{G}$ has weight 1=w−2, we first reversely traverse the graph starting from $TCT_{G}$ to reach its only predecessor $v^{\prime }=GTC$, which has weight 2=w−1. Then, the weight representations of v and $v^{\prime }$ read as 100 *and *101*, respectively. We then apply the proof steps in [6] Lemma 2.2 and [6] Corollary 2.3 to construct a path P from *100 *to *101 *on the binary version of the de Bruijn graph $D(B_{2}^{3}\left(4\right))$. It turns out that this path P induces the sequence *1001101 on $D(B_{2}^{3}\left(4\right))$, and by an abuse of notation we write P=1001101. Based on P, we find that the path $P^{\prime }=CAAGCAC$ is a valid path on $G_{0}$ from $v=CAA_{G}$ to $v^{\prime \prime }=CAC$. More explicitly, $P^{\prime }$ traverses in the following order: $CAA_{G}\to AAG_{C}\to AGC\to GCA\to CAC$. Note that the weight representation of $v^{\prime \prime }=CAC$ is the same as that of $v^{\prime }=GTC$. Then, we can initiate a walk from $v^{\prime \prime }=CAC$ to $v^{\prime }=GTC$ following the path CACGTC on $G_{0}$. These arguments shows that from v we can reach $v^{*}$ through $v\to v^{\prime \prime }\to v^{\prime }\to v^{*}$.

## 6. Generalized Orthogonal Kautz Sequences

Next, we study possible extensions of the concepts of generalized orthogonal de Bruijn sequences to Kautz sequences. More explicitly, we introduce the notions of *ℓ*-orthogonal Kautz sequences, orthogonal balanced Kautz sequences, and orthogonal fixed-weight Kautz sequences. We start by recalling the definition of Kautz sequences.

**Definition 10.**  *Let k≥1, and let A be an alphabet of size σ≥3. Define $K_{k}\left(A\right)$ to be the collection of all sequences in $A^{k}$ that do not have two or more adjacent identical characters (i.e., no run lengths/homopolymers of length at least two). Then, a circular sequence $s=(s_{0},s_{1},\ldots ,s_{\sigma {\left(\sigma -1\right)}^{k-1}-1})$ is called a* (σ,k)-Kautz sequence *if each sequence in $K_{k}\left(A\right)$ appears as a circular substring of s exactly once.*

**Remark 4.**  *Using the terminology in Definition 8, a Kautz sequence can be interpreted as a de Bruijn sequence with respect to the set $K_{k}\left(A\right)$.*

Similar to de Bruijn sequences, Kautz sequences are closely related to *Kautz graphs*. Using the notation in Definition 9, a Kautz graph $G_{\sigma ,k}^{\mathrm{Kautz}}:=D\left(K_{k}\left(A\right)\right)$ is a generalized de Bruijn graph with respect to the set $K_{k}\left(A\right)$. Consequently, there is a one-to-one correspondence between the (σ,k)-Kautz sequences and the Eulerian circuits on $G_{\sigma ,k}^{\mathrm{Kautz}}$ [8]. In addition, there is also a one-to-one correspondence between the (σ,k)-Kautz sequences and the Hamiltonian cycles on $G_{\sigma ,k+1}^{\mathrm{Kautz}}$ [8].

**Figure 4:**
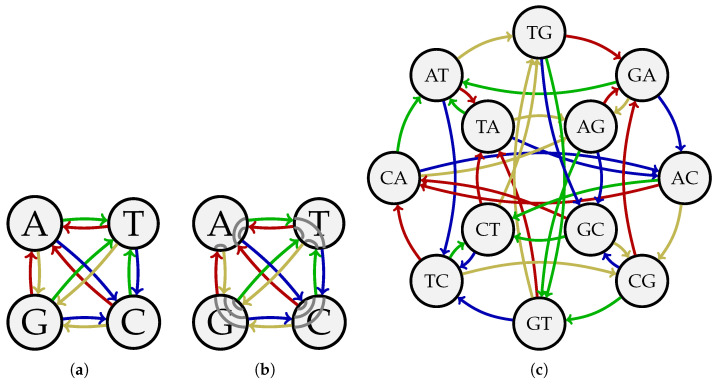
(**a**) The Kautz graph $G_{4,2}^{\mathrm{Kautz}}$. (**b**) The wiring of $G_{4,2}^{\mathrm{Kautz}}$ induced by the Eulerian circuit corresponding to the (4,2)-Kautz sequence ATCGAGCTGTAC. (**c**) The Kautz graph $G_{4,3}^{\mathrm{Kautz}}$.

**Example 6.**  *Consider the DNA alphabet A={A,T,C,G} and k=2. The sequence s=ATCGAGCTGTAC is a (4,2)-Kautz sequence since each length*-2 *sequence of unequal symbols from A appears as a circular substring of s exactly once. Figure 4a depicts the Kautz graph $G_{4,2}^{\mathrm{Kautz}}$, which has an Eulerian circuit corresponding to s. The wiring induced by that circuit is illustrated in Figure 4b. The sequence s can also be represented by a Hamiltonian cycle on the Kautz graph $G_{4,3}^{\mathrm{Kautz}}$ in Figure 4c. This cycle is given by AT→TC→CG→GA→AG→GC→CT→TG→GT→TA→AC→CA→AT.*

### 6.1. *ℓ*-Orthogonal Kautz Sequences

By generalizing Definition 4, we describe the notion of *ℓ*-orthogonality of Kautz sequences as follows. A collection of (σ,k)-Kautz sequences $C=\{s_{1},\ldots ,s_{M}\}$ is called *ℓ*-orthogonal if each sequence in $K_{k+1}\left(A\right)$ appears at most *ℓ* times in C, where ℓ≥1 and A is an alphabet of size σ. Similar to the case of de Bruijn sequences, for any collection C of (σ,k)-Kautz sequences, the following statements are equivalent:

- C is *ℓ*-orthogonal.
- The corresponding Eulerian circuits on $G_{\sigma ,k}^{\mathrm{Kautz}}$ use each pair of consecutive arcs at most *ℓ* times.
- The corresponding Hamiltonian cycles on $G_{\sigma ,k+1}^{\mathrm{Kautz}}$ use each arc at most *ℓ* times.

Let $\Omega_{\ell }^{\mathrm{Kautz}}\left(\sigma ,k\right)$ be the maximum cardinality of any collection of *ℓ*-orthogonal (σ,k)-Kautz sequences. By adapting the proofs of Proposition 1 and Theorem 1, we can bound $\Omega_{\ell }^{\mathrm{Kautz}}\left(\sigma ,k\right)$ as follows.

**Proposition 4.**  *We have $\Omega_{\ell }^{\mathrm{Kautz}}\left(\sigma ,k\right)\leq \ell \left(\sigma -1\right)$. If $\ell \leq \sigma {\left(\sigma -1\right)}^{k-2}$ and σ≥4, then we further have $\Omega_{\ell }^{\mathrm{Kautz}}\left(\sigma ,k\right)\geq 2\ell$ and $\Omega_{\ell }^{\mathrm{Kautz}}\left(\sigma ,k\right)\geq \ell \left\lfloor \left(\sigma -1\right)/2\right\rfloor$.*

**Sketch of the proof.**  First observe that every vertex in the Kautz graph $G_{\sigma ,k}^{\mathrm{Kautz}}$ has σ−1 incoming arcs, σ−1 outgoing arcs, and no loop. The same arguments in the proof of Proposition 1 proves the upper bound on $\Omega_{\ell }^{\mathrm{Kautz}}\left(\sigma ,k\right)$.

Furthermore, note that by adapting the proof of Theorem 1, we actually can establish the following claim: For any Eulerian directed graph *G* with minimum in/out-degree δ and any ℓ≤|V(G)|, *G* has a collection of ℓK Eulerian circuits such that each pair of consecutive arcs appears in this collection at most *ℓ* times, where K=2 for δ=3 and K=⌊δ/2⌋ for δ≥4. The lower bounds on $\Omega_{\ell }^{\mathrm{Kautz}}\left(\sigma ,k\right)$ can then be immediately deduced by the fact that the minimum in/out-degree of $G_{\sigma ,k}^{\mathrm{Kautz}}$ is σ−1. □

**Example 7.**  *We verify that the lower bound of $\Omega_{\ell }^{\mathrm{Kautz}}\left(\sigma ,k\right)$ in Proposition 4 holds for σ=4, k=2, and ℓ=2. Setting these parameters in Proposition 4 gives $\Omega_{\ell }^{\mathrm{Kautz}}\left(\sigma ,k\right)\geq 2\ell =4$. Thus, we seek to find a collection of four (4,2)-Kautz sequences such that each sequence in $K_{3}\left(A\right)$ appears at most twice in C. Following the proof of Theorem 1, we partition $V(G_{4,2})$ into $V_{1}=\left\{A,T\right\}$ and $V_{2}=\left\{C,G\right\}$ and start with the Eulerian circuit $C_{1,1}=ATCGAGCTGTAC$ on $G_{4,2}$. After the rewiring process, we obtain the following three (4,2)-Kautz sequences:*

$$\begin{array}{ccc} \; & C_{1,2}=ACAGCTATGTCG,\; & C_{2,1}=ACTATGCGTCAG,\\ \; & C_{2,2}=ACTGCGTAGATC. \end{array}$$

*It can be checked that the collection $C=\{C_{1,1},C_{1,2},C_{2,1},C_{2,2}\}$ is *2*-orthogonal. For example, the string ATC appears twice (in $C_{1,1}$ and in $C_{2,2}$), while the string GAG appears once (in $C_{1,1}$), and the string ATA does not appear at all.*

### 6.2. Orthogonal Balanced Kautz Sequences

We now generalize Definition 3 in order to define balanced Kautz sequences as follows.

**Definition 11.**  *Let k and A be as before, and let b≥1. We say that a circular sequence $s=(s_{0},s_{1},\ldots ,s_{b\sigma {\left(\sigma -1\right)}^{k-1}-1})$ is a b-balanced* (σ,k)-Kautz sequence *if each sequence in $K_{k}\left(A\right)$ appears as a circular substring of s exactly b times.*

The following terminology is similar to that used in Section 4. We say that a collection C of *b*-balanced (σ,k)-Kautz sequences is *orthogonal* if each sequence in $K_{k+1}\left(A\right)$ appears at most once in C. A necessary condition for a (σ,k)-Kautz sequence s to belong to an orthogonal collection is that s itself contains no sequence in $K_{k+1}\left(A\right)$ more than once. In this case, we say that s is self-orthogonal. Then, there is a one-to-one correspondence between self-orthogonal *b*-balanced (σ,k)-Kautz sequences and *b*-circuits on $G_{\sigma ,k+1}^{\mathrm{Kautz}}$, where the *b*-circuits are defined as in Definition 7.

For c≥2, we define $\Sigma^{\mathrm{Kautz}}\left(c,b,k\right)$ to be the smallest σ such that there exist *c* orthogonal *b*-balanced (σ,k)-Kautz sequences. We have the following bounds on $\Sigma^{\mathrm{Kautz}}\left(c,b,k\right)$:

**Proposition 5.**  *Assume b≥2 and c≥2. Then we have $cb+1\leq \Sigma^{\mathrm{Kautz}}\left(c,b,k\right)\leq 2cb+1$.*

**Sketch of the proof.**  The proof of the lower bound on $\Sigma^{\mathrm{Kautz}}\left(c,b,k\right)$ is similar to that of Proposition 3 and is omitted.

To prove the upper bound, it suffices to prove that $G_{2cb+1,k}^{\mathrm{Kautz}}$ has *c* arc-disjoint *b*-circuits. We first invoke Lemma 2 to deduce that there are ⌊(2cb+1−1)/2⌋=cb pairwise compatible Eulerian circuits on $G_{2cb+1,k}^{\mathrm{Kautz}}$. These circuits correspond to cb arc-disjoint Hamiltonian cycles on $G_{2cb+1,k}^{\mathrm{Kautz}}$. Then, we can arbitrarily partition the collection of these cycles into *c* groups, each having *b* cycles. The combination of the cycles in each group is a *b*-circuit on $G_{2cb+1,k}^{\mathrm{Kautz}}$, and the *b*-circuits combined from different groups are arc-disjoint. Therefore, $G_{2cb+1,k}^{\mathrm{Kautz}}$ has *c* arc-disjoint *b*-circuits, which proves the upper bound on $\Sigma^{\mathrm{Kautz}}\left(c,b,k\right)$. □

**Remark 5.**  *Since de Bruijn sequences are also characterized by both Eulerian circuits and Hamiltonian cycles, applying the same steps in the proof of Proposition 5 gives Σ(c,b,k)≤2cb, where Σ(c,b,k) is defined in Section 4. However, Theorem 2 always gives an upper bound of Σ(c,b,k) strictly smaller than 2cb since there is always a power of two contained in [cb,2cb−1].*

### 6.3. Orthogonal Fixed-Weight Kautz Sequences

We first define fixed-weight Kautz sequences. Following the definitions in Section 5, consider the case A=W∪X with each symbol in W having weight 1 and each symbol in X having zero weight. For $0\leq w^{\prime }\leq w\leq k$, we then define a fixed-weight Kautz sequence with parameters $\left(|A|,k,w^{\prime },w\right)$ as a de Bruijn sequence with respect to $K_{k}\left(A\right)\cap A_{w^{\prime }}^{w}\left(k\right)$. We have the following conditions on the existence of such sequences:

**Proposition 6.**  *A fixed-weight Kautz sequence with parameters $\left(|A|,k,w^{\prime },w\right)$ exists if and only if $w^{\prime }$ and w satisfy one of the following conditions:*

*1.*  *$w^{\prime }=w=0$;*
*2.*  *$w^{\prime }=w=k$;*
*3.*  *$w^{\prime }\in \left\{0,1\right\}$ and w∈{k−1,k}.*

**Proof.**  We first prove that the conditions in Proposition 6 imply the existence of a fixed-weight Kautz sequence with associated parameters. If $w^{\prime }=w=0$, then $K_{k}\left(A\right)\cap A_{w^{\prime }}^{w}\left(k\right)=K_{k}\left(X\right)$, and the fixed-weight sequences are simply the (|X|,k)-Kautz sequences. Similarly, the case $w^{\prime }=w=k$, corresponds to (|W|,k)-Kautz sequences. Among the four subcases with $w^{\prime }\in \left\{0,1\right\}$ and w∈{k−1,k}, the subcase $\left(w^{\prime },w\right)=\left(0,k\right)$ corresponds to (|A|,k)-Kautz sequences since $K_{k}\left(A\right)\cap A_{w^{\prime }}^{w}\left(k\right)=K_{k}\left(A\right)$. The remaining three subcases $\left(w^{\prime },w\right)=\left(0,k-1\right)$, $\left(w^{\prime },w\right)=\left(1,k-1\right)$, and $\left(w^{\prime },w\right)=\left(1,k\right)$ can be seen as a direct consequence of Proposition 7, which we will state and prove later.

Now, assume none of the conditions in Proposition 6 hold. We seek to show that the generalized de Bruijn graph $D(K_{k}\left(A\right)\cap A_{w^{\prime }}^{w}\left(k\right))$ is not Eulerian. Since Condition 3 fails to hold, we have either $w^{\prime }\geq 2$ or w≤k−2. First assume $w^{\prime }\geq 2$. Since Condition 2 fails to hold and $w^{\prime }\leq w$, we can assume $w^{\prime }\leq k-1$. Now, note that the vertex set of $D(K_{k}\left(A\right)\cap A_{w^{\prime }}^{w}\left(k\right))$ is $V\left(D\left(K_{k}\left(A\right)\cap A_{w^{\prime }}^{w}\left(k\right)\right)\right)=K_{k-1}\left(A\right)\cap A_{w^{\prime }-1}^{w}\left(k-1\right)$. Thus, we can pick a vertex v with weight $w^{\prime }-1$ in the form of $v=(a_{0},a_{1},\ldots ,a_{w^{\prime }-2},b_{w^{\prime }-1},\ldots b_{k-2})$, where $a_{0},a_{1}\ldots ,a_{w^{\prime }-2}\in W$ and $b_{w^{\prime }-1},\ldots ,b_{k-2}\in X$. Since v has weight $w^{\prime }-1$, each of its predecessors must start with a weight-1 symbol. Then note that v starts with $a_{0}\in W$ and no vertex can have two adjacent identical symbol, v has |W|−1 predecessors. Similarly, each of its successors must end with a weight-1 symbol, but since v ends with $b_{k-2}\in X$, v has |W| successors. Therefore, the in-degree of v is not equal to its out-degree, which implies that $D(K_{k}\left(A\right)\cap A_{w^{\prime }}^{w}\left(k\right))$ is not Eulerian.

The case w≤k−2 is handled similarly. Since Condition 1 fails to hold and $w^{\prime }\leq w$, we can assume w≥1. Then we can pick a vertex $v^{\prime }$ with weight *w* in the form of $v^{\prime }=\left(a_{0},a_{1},\ldots ,a_{w-1},b_{w},\ldots ,b_{k-2}\right)$, where $a_{0},a_{1}\ldots ,a_{w-1}\in W$ and $b_{w},\ldots ,b_{k-2}\in X$. A similar argument shows that $v^{\prime }$ has in-degree |X| and out-degree |X|−1, which are not equal. □

**Figure 5:**
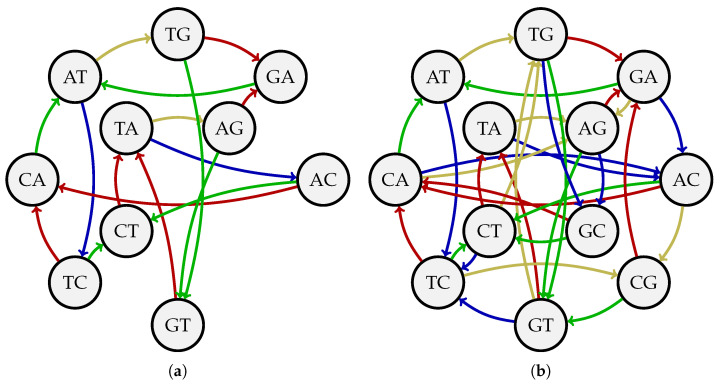
Some fixed-weight Kautz graphs with alphabet A={A,T,C,G} and k=3: (**a**) The fixed-weight Kautz graph $D(K_{3}\left(A\right)\cap A_{1}^{1}\left(3\right))$. (**b**) The fixed-weight Kautz graph $D(K_{3}\left(A\right)\cap A_{1}^{2}\left(3\right))$.

**Example 8.**  *Let A={A,T,C,G} and k=3. First consider $w^{\prime }=w=1$, which fails to meet any of the three conditions in Proposition 6. Figure 5a shows the fixed-weight Kautz graph $D(K_{3}\left(A\right)\cap A_{1}^{1}\left(3\right))$. It can be seen that $D(K_{3}\left(A\right)\cap A_{1}^{1}\left(3\right))$ is not Eulerian since the vertex CA has one successor AT and two predecessors, AC and TC. As a result, no fixed-weight Kautz sequence with parameters $(|A|,k,w^{\prime },w)=\left(4,3,1,1\right)$ exists. This observation is consistent with Proposition 6.*

*Next, consider the case $w^{\prime }=1$ and w=2, which satisfies Condition 3 in Proposition 6. The fixed-weight Kautz graph $D(K_{3}\left(A\right)\cap A_{1}^{2}\left(3\right))$ is shown in Figure 5b. It can be verified that the sequence s=CAGATCATGACACTACGAGTAGCTCTGTCGTG can be represented by an Eulerian circuit on $D(K_{3}\left(A\right)\cap A_{1}^{2}\left(3\right))$. Thus s is a fixed-weight Kautz sequence with parameters $(|A|,k,w^{\prime },w)=\left(4,3,1,2\right)$. This result agrees with Proposition 6.*

Orthogonality of fixed-weight Kautz sequences with $\left(w^{\prime },w\right)=\left(0,0\right)$, (k,k), or (0,k) can be deduced from the orthogonality of ordinary Kautz sequences, as explained in [8]. For other allowed cases of $\left(w^{\prime },w\right)$ in Proposition 6, we have the following results.

**Proposition 7.**  *Assume that |W|≥2 and |X|≥2. Let δ:=|W|+|X|−1 denote the minimum in/out-degree of the ordinary Kautz graph $G_{\sigma ,k}^{\mathrm{Kautz}}$. We have the following:*

- *$D(K_{k}\left(A\right)\cap A_{1}^{k-1}\left(k\right))$ has min(|W|,|X|,⌊δ/2⌋) pairwise compatible Eulerian circuits.*
- *$D(K_{k}\left(A\right)\cap A_{0}^{k-1}\left(k\right))$ has min(|X|,⌊δ/2⌋) pairwise compatible Eulerian circuits.*
- *$D(K_{k}\left(A\right)\cap A_{1}^{k}\left(k\right))$ has min(|W|,⌊δ/2⌋) pairwise compatible Eulerian circuits.*

**Sketch of the proof.**  We only prove the result for the case of $D(K_{k}\left(A\right)\cap A_{1}^{k-1}\left(k\right))$, since the other two cases can be handled similarly.

This proof is similar to that of Theorem 3. Write $G:=D(K_{k}\left(A\right)\cap A_{1}^{k-1}\left(k\right))$ for simplicity. The vertex set of *G* is $V\left(G\right)=K_{k-1}\left(A\right)$. Any vertex in *G* with weight 0 has in/out-degree |W|, any vertex with weight k−1 has in/out-degree |X|, and any other vertex has in/out-degree δ. We apply the same vertex-splitting technique in the proof of Theorem 3. For each vertex v with weight 0 or k−1, there exist min(|W|,|X|) edge-disjoint wirings $W_{1}\left(v\right),\ldots ,W_{\min(|W|,|X|)}\left(v\right)$ of v. Define the split graphs $G_{1},\ldots ,G_{\min(|W|,|X|)}$ as follows: $G_{j}$ is obtained by splitting each vertex v with weight 0 or k−1 along its *j*th wiring $W_{j}\left(v\right)$. Then, as long as each split graph $G_{j}$ is Eulerian, we can select min(|W|,|X|,⌊δ/2⌋) of them, merge each one back, and then rewire each remaining vertex with weight in [1,k−2] one by one via Lemma 2. This process gives min(|W|,|X|,⌊δ/2⌋) pairwise compatible Eulerian circuits.

To show that each split graph $G_{j}$ is Eulerian, first note that each split vertex has in/out-degree 1 and each unmodified vertex has in/out-degree δ. Thus, each vertex in $G_{j}$ has equal in-degree and out-degree. Then, we show that $G_{j}$ is strongly connected. Note that it suffices to show that the subgraph *H* of $G_{j}$ obtained by removing all the split vertices is strongly connected since each split vertex can be either forwardly or reversely traversed to one vertex in *H*. It is noteworthy that *H* is the same for all $G_{j}$’s and is identical to the subgraph of $G_{\sigma ,k}^{\mathrm{Kautz}}$ obtained by removing all the vertices with weight 0 or k−1.

Let $v=(v_{0},\ldots ,v_{k-2})$ and $v^{\prime }=\left(v_{0}^{\prime },\ldots ,v_{k-2}^{\prime }\right)$ be two vertices in *H*. Since the weight of v is at least 1, there must be a symbol in v that is weighted. That is, there exists some t∈[0,k−2] such that $v_{t}\in W$. Now consider the walk *P* on $G_{\sigma ,k}^{\mathrm{Kautz}}$ from v to $\hat{v}=\left(v_{t+1},\ldots ,v_{k-2},{\hat{v}}_{0},\ldots ,{\hat{v}}_{t}\right)$ that induces the sequence $\left(v_{0},\ldots ,v_{k-2},{\hat{v}}_{0},\ldots ,{\hat{v}}_{t}\right)$, where ${\hat{v}}_{0},\ldots ,{\hat{v}}_{t}$ are defined as follows:

- If $v_{k-2}\neq v_{0}$, then we choose ${\hat{v}}_{s}=v_{s}$ for s∈[0,t].
- If $v_{k-2}=v_{0}\;\in W$, we first choose a permutation π on W such that $v_{0}$ is not fixed by π. In other words, π is a bijection from W to itself satisfying $\pi \left(v_{0}\right)\neq v_{0}$. Note that such a permutation π always exists since we assumed that |W|≥2. Then for s∈[0,t], define
  $$\begin{array}{c} {\hat{v}}_{s}:=\left\{\begin{array}{cc} \pi (v_{s}), & \mathrm{if}\;v_{s}\in W,\\ v_{s}, & \mathrm{if}\;v_{s}\in X. \end{array}\right. \end{array}$$
- If $v_{k-2}=v_{0}\in X$, for s∈[0,t] we similarly define
  $$\begin{array}{c} {\hat{v}}_{s}:=\left\{\begin{array}{cc} v_{s}, & \mathrm{if}\;v_{s}\in W,\\ \tau (v_{s}), & \mathrm{if}\;v_{s}\in X, \end{array}\right. \end{array}$$
  where τ is a permutation on X that does not fix $v_{0}$, i.e., $\tau \left(v_{0}\right)\neq v_{0}$.

It can be seen that no two adjacent symbols in the sequence $\left(v_{0},\ldots ,v_{k-2},{\hat{v}}_{0},\ldots ,{\hat{v}}_{t}\right)$ are the same, and thus the walk *P* is indeed a valid walk on $G_{\sigma ,k}^{\mathrm{Kautz}}$. Furthermore, the weight representation of the sequence $\left(v_{0},\ldots ,v_{k-2},{\hat{v}}_{0},\ldots ,{\hat{v}}_{t}\right)$ is the same as $\left(v_{0},\cdots ,v_{k-2},v_{0},\ldots ,v_{t}\right)$. This implies that the walk *P* only visits vertices having the same weight as v. Therefore, *P* is also a valid walk in the subgraph *H*.

Similarly, since the weight of $v^{\prime }$ is at most k−2, there exists some u∈[0,k−2] such that $v_{u}^{\prime }\in X$. Then, consider the vertex ${\hat{v}}^{\prime }=\left({\hat{v}}_{u}^{\prime },\ldots ,{\hat{v}}_{k-2}^{\prime },v_{0}^{\prime },\ldots ,v_{u-1}^{\prime }\right)$, where ${\hat{v}}_{u}^{\prime },\ldots ,{\hat{v}}_{k-2}^{\prime }$ are defined in a similar way as follows:

- If $v_{0}^{\prime }\neq v_{k-2}^{\prime }$, define ${\hat{v}}_{r}^{\prime }=v_{r}^{\prime }$ for r∈[u,k−2].
- If $v_{0}^{\prime }=v_{k-2}^{\prime }\;\in W$, find a permutation $\pi^{\prime }$ on W that does not fix $v_{0}^{\prime }$. Then for r∈[u,k−2], we define
  $$\begin{array}{c} {\hat{v}}_{r}^{\prime }:=\left\{\begin{array}{cc} \pi^{\prime }\left(v_{r}^{\prime }\right), & \mathrm{if}\;v_{r}^{\prime }\in W,\\ v_{r}^{\prime }, & \mathrm{if}\;v_{r}^{\prime }\in X. \end{array}\right. \end{array}$$
- If $v_{0}^{\prime }=v_{k-2}^{\prime }\in X$, for r∈[u,k−2] we define
  $$\begin{array}{c} {\hat{v}}_{r}^{\prime }:=\left\{\begin{array}{cc} v_{r}^{\prime }, & \mathrm{if}\;v_{r}^{\prime }\in W,\\ \tau^{\prime }\left(v_{r}^{\prime }\right), & \mathrm{if}\;v_{r}^{\prime }\in X, \end{array}\right. \end{array}$$
  where $\tau^{\prime }$ is a permutation on X that does not fix $v_{0}^{\prime }$.

It follows that the walk from ${\hat{v}}^{\prime }$ to $v^{\prime }$ that induces the sequence $\left({\hat{v}}_{u}^{\prime },\ldots ,{\hat{v}}_{k-2}^{\prime },v_{0}^{\prime },\ldots v_{k-2}^{\prime }\right)$ is a valid walk in *H*. Then, note that we have ${\hat{v}}_{t}\in W$ since $v_{t}\in W$ and ${\hat{v}}_{t}$ is either $v_{t}$ or $\pi (v_{t})$. Similarly, ${\hat{v}}_{u}^{\prime }\in X$. In particular, we have ${\hat{v}}_{t}\neq {\hat{v}}_{u}^{\prime }$. Thus, we can walk in $G_{\sigma ,k}^{\mathrm{Kautz}}$ from $\hat{v}$ to ${\hat{v}}^{\prime }$ inducing the sequence $\left(v_{t+1},\ldots ,v_{k-2},{\hat{v}}_{1},\ldots ,{\hat{v}}_{t},{\hat{v}}_{u}^{\prime },\ldots ,{\hat{v}}_{k-2}^{\prime },v_{0}^{\prime }\ldots ,v_{u-1}^{\prime }\right)$. Denote this walk by $\bar{P}$. Furthermore, any vertex visited by $\bar{P}$ contains the substring ${\hat{v}}_{t}{\hat{v}}_{u}^{\prime }$. Recall that ${\hat{v}}_{t}$ has weight 1 and ${\hat{v}}_{u}^{\prime }$ has weight 0. As a result, any vertex visited by $\bar{P}$ must have weight between 2 and k−2, implying that the whole walk $\bar{P}$ lies in *H*. In conclusion, we can walk in *H* from v to $\hat{v}$, then from $\hat{v}$ to ${\hat{v}}^{\prime }$, and then from ${\hat{v}}^{\prime }$ to $v^{\prime }$. These arguments demonstrate that *H* is strongly connected, and thus so is each split graph $G_{j}$.

These arguments prove the statement of the result. □

## 7. Conclusions

Motivated by applications in genetic sequence screening and DNA-based data storage, we generalized the problem of counting orthogonal order-*k* de Bruijn sequences. In particular, we relaxed the orthogonality constraint by allowing multiple repeats of the (k+1)-substrings across the sequences; we extended the notion of de Bruijn sequences to balanced de Bruijn sequences, and then examined the notion of orthogonal balanced de Bruijn sequences. In both cases, we derived upper and lower bounds on the size of the set of sequences and repeated similar derivations for the closely related family of Kautz sequences.

## Data Availability

The original contributions presented in this study are included in the article. Further inquiries can be directed to the corresponding author.
